# Urinary concentrations of the soluble adhesion molecule E-cadherin and total protein in patients with bladder cancer

**DOI:** 10.1038/sj.bjc.6690351

**Published:** 1999-04

**Authors:** A S Protheroe, R E Banks, M Mzimba, W H Porter, J Southgate, P N Singh, M Bosomworth, P Harnden, P H Smith, P Whelan, P J Selby

**Affiliations:** Imperial Cancer Research Fund Cancer Medicine Research Unit, St James's University Hospital, Leeds LS9 7TF, UK; Departments of Urology, Chemical Pathology, and Pathology, St James's University Hospital, Leeds LS9 7TF, UK; Leeds General Infirmary, Leeds LS29JT, UK

**Keywords:** soluble adhesion molecules, E-cadherin, bladder, urine, cancer

## Abstract

Reduced expression of the adhesion molecule E-cadherin has been associated with increased invasiveness and poorer survival in patients with bladder cancer. We have examined soluble E-cadherin (sE-cadherin) and total protein concentrations in urine from patients with bladder cancer (*n* = 34), non-neoplastic benign urological diseases (*n* = 14) and healthy controls (*n* = 21) to determine their diagnostic and prognostic significance. Soluble E-cadherin concentrations of the cancer group were significantly higher (*P* < 0.001) than those of the controls but the benign group was not significantly different from either the cancer group or the controls. When sE-cadherin concentrations were adjusted for creatinine, similar but more statistically significant results were obtained and the benign group was significantly elevated compared with the controls (*P* < 0.01). No differences were apparent between the invasive (pT1–4) and non-invasive (pTa) cancers. Urinary total protein concentrations in the cancer group were significantly higher than the controls (*P* < 0.001) and the benign group (*P* < 0.05) although no difference was seen between the benign group and patients with non-invasive (pTa) cancer or between the benign group and controls. When expressed as the protein/creatinine index, results were similar but more statistically significant and a significant difference was seen between invasive and non-invasive cancers (*P* < 0.01). Only the protein/creatinine index correlated significantly with stage of the tumour (*P* < 0.01). It is concluded that urinary sE-cadherin measurements are of no greater value than urinary total protein. © 1999 Cancer Research Campaign


*British Journal of Cancer* (1999) **80**(1/2), 273–278
© 1999 Cancer Research Campaign
Article no. bjoc.1998.0351

### Urinary concentrations of the soluble adhesion molecule E-cadherin and total protein in patients with bladder cancer

**AS Protheroe[1], RE Banks[1], M Mzimba[1], WH Porter*, J Southgate[1], PN Singh[2], M Bosomworth[4,5], P Harnden[4,5], PH Smith[2], P Whelan[2] and PJ Selby[1]**

[1]Imperial Cancer Research Fund Cancer Medicine Research Unit, St James's University Hospital, Leeds LS9 7TF, UK; Departments of [2]Urology, [3]Chemical Pathology and [4]Pathology, St James's University Hospital, Leeds LS9 7TF, UK; [5]Leeds General Infirmary, Leeds LS2 9JT, UK

**Summary** Reduced expression of the adhesion molecule E-cadherin has been associated with increased invasiveness and poorer survival in patients with bladder cancer. We have examined soluble E-cadherin (sE-cadherin) and total protein concentrations in urine from patients with bladder cancer ($n$ = 34), non-neoplastic benign urological diseases ($n$ = 14) and healthy controls ($n$ = 21) to determine their diagnostic and prognostic significance. Soluble E-cadherin concentrations of the cancer group were significantly higher ($P < 0.001$) than those of the controls but the benign group was not significantly different from either the cancer group or the controls. When sE-cadherin concentrations were adjusted for creatinine, similar but more statistically significant results were obtained and the benign group was significantly elevated compared with the controls ($P < 0.01$). No differences were apparent between the invasive (pT1–4) and non-invasive (pTa) cancers. Urinary total protein concentrations in the cancer group were significantly higher than the controls ($P < 0.001$) and the benign group ($P < 0.05$) although no difference was seen between the benign group and patients with non-invasive (pTa) cancer or between the benign group and controls. When expressed as the protein/creatinine index, results were similar but more statistically significant and a significant difference was seen between invasive and non-invasive cancers ($P < 0.01$). Only the protein/creatinine index correlated significantly with stage of the tumour ($P < 0.01$). It is concluded that urinary sE-cadherin measurements are of no greater value than urinary total protein.

**Keywords:** soluble adhesion molecules; E-cadherin; bladder; urine; cancer

Bladder cancer is the fourth most common cancer in men and the tenth in women, with approximately 12 500 new cases in the UK (Cancer Research Campaign Factsheet 9.1, 1997). Stage and grade as measures of the depth of invasion into the bladder wall and degree of differentiation of the tumour, respectively, are currently the most widely used indicators of prognosis. Other known adverse risk factors include the presence of multiple tumours at presentation (Parmar et al, 1989), the recurrence of tumours at 3 months post-cystoscopy (Reading et al, 1995) and p53 mutations (Esrig et al, 1994; Caliskan et al, 1997).

For patients presenting with superficial bladder cancer (pTa and pT1) the prognosis is good. Although recurrences occur in approximately 70% of patients, only 10–15% progress to muscle-invasive disease (Hudson and Catalona, 1996). However, there is a need for regular monitoring to identify patients at risk of recurrence or progression as early as possible so that appropriate therapy can be initiated promptly. To date, there are no established tumour markers available, although several have been investigated in preliminary studies with promising results in terms of diagnostic or prognostic use. These include bladder-tumour associated analyte (BTA; Sarosdy et al, 1995; D'Hallewin and Baert, 1996; Leyh et al, 1997), nuclear matrix protein 22 (NMP22; Soloway et al, 1996), telomerase activity (Kinoshita et al, 1997; Yoshida et al, 1997), Ki-67 antigen and p53 protein expression (Tsuji et al, 1997), microsatellite analysis (Mao et al, 1996; Steiner et al, 1997) and matrix metalloproteinases, their inhibitors and activators (Gohji et al, 1998; Kanayama et al, 1998).

Cadherins are $Ca^{2+}$-dependent homotypic adhesion molecules essential for maintaining intercellular adhesion (Takeichi, 1990). E-cadherin (120 kDa) is mainly involved in epithelial cell–cell interactions with loss being associated with increased invasiveness and decreased differentiation in a variety of malignancies including breast, prostate and gastric carcinoma (Takeichi, 1993). Decreased, heterogeneous or absent expression of membrane-associated E-cadherin has been reported in malignant bladder tumours, with abnormal expression correlating with increasing grade and invasiveness (Bringuier et al, 1993; Otto et al, 1994; Lipponen and Eskelinen, 1995; Ross et al, 1995; Syrigos et al, 1995) and associated with reduced survival (Bringuier et al, 1993; Syrigos et al, 1995). Similarly in patients with pTa or pT1 bladder tumours, abnormal expression of E-cadherin has been associated with more rapid progression and reduced survival (Otto et al, 1994; Lipponen and Eskelinen 1995).

Although changes in expression of E-cadherin may account for changes in cell invasiveness, additional mechanisms may also contribute to altered function of this adhesion molecule (Hiraguri et al, 1998). Point mutations in the calcium binding sites (Ozawa

*Received 27 January 1998*
*Revised 3 October 1998*
*Accepted 21 October 1998*

*Correspondence to:* RE Banks

*On sabbatical leave from the Department of Pathology and Laboratory Medicine, University of Kentucky Medical Center, USA

et al, 1990; Kanai et al, 1994), loss of heterozygosity (Becker and Hofler, 1995), hypermethylation of the promoter region (Yoshuira et al, 1995), aberrant function of the catenins (Shimoyama et al, 1992) or increased shedding of E-cadherin from the cell membrane (Matsuura et al, 1992), have all been proposed as contributory mechanisms for impairment of adhesive function.

Soluble forms of several adhesion molecules have been identified (Gearing and Newman, 1993), and a soluble form of E-cadherin (sE-cadherin; 80 kDa) has been shown to be shed by the MCF-7 breast cancer line (Damsky et al, 1983). Proteolytic cleavage of E-cadherin from the cell surface may account for the soluble form (Matsuura et al, 1992), which has been found in serum (Katayama et al, 1994*a*; Griffiths et al, 1996) and in urine (Katayama et al, 1994*b*; Banks et al, 1995) with elevated levels in patients with a variety of cancers. Following the recent finding of elevated serum sE-cadherin concentrations in patients with bladder tumours (Griffiths et al, 1996), we have performed a preliminary cross-sectional study measuring urinary sE-cadherin levels in patients with bladder cancer, non-neoplastic conditions and healthy volunteers and compared these with urinary total protein levels to establish whether such measurements may be of use clinically for diagnostic or monitoring purposes.

### MATERIALS AND METHODS

Urine samples were collected over a period of several months from patients attending the urology ward for investigations prior to treatment. Details of the patient and control groups are provided in Table 1. Aprotinin was added (final concentration 100 KIU $ml^{-1}$) and the samples were centrifuged at 1500 ***g*** for 10 min to remove cellular debris before being aliquoted and stored at –70°C until assayed. Additional samples were collected from healthy volunteers. Histological specimens were reviewed independently and blindly by a single pathologist and all tumour sections were staged according to the TNM classification (Hermanek and Sobin, 1987) and graded according to the WHO criteria (Mostofi et al, 1973). The tumour stage and grade of the patients with bladder cancer are shown in Table 2. The benign group consisted of two patients with inflammatory conditions of the bladder, four with benign prostatic hypertrophy, two with kidney stones, one with moderate dysplasia and five were patients who had previously been treated for non-invasive malignancies but currently had normal biopsies.

Soluble E-cadherin concentrations were determined using a commercially available enzyme-linked immunosorbent assay (ELISA) according to the manufacturer's instructions (Takara Shuzo, Japan). This assay is based on the capture of sE-cadherin using a solid-phase adsorbed monoclonal antibody followed by subsequent detection using a labelled second monoclonal antibody to E-cadherin, with a detection limit of < 50 ng $ml^{-1}$. Urinary protein concentrations were determined by standard automated analysis with a Bayer Axon analyser using pyrogallol red reagent (0.05 mM; Sigma, UK) with a timed end-point method and standardization against human albumin (Sigma). Creatinine concentrations in urine were measured by the Jaffe method (Rock et al, 1986) using the Bayer Axon analyser. Both sE-cadherin and total protein concentrations were adjusted for creatinine concentration and expressed as a sE-cadherin/creatinine index or protein/creatinine index, respectively, calculated as protein (g $l^{-1}$) × 10 000 divided by creatinine (mmol $l^{-1}$) with units of × 10 g $\mu mol^{-1}$ to be in accordance with previous studies (Dyson et al, 1992; Mitchell et al, 1993).

Urine samples from 12 controls and 21 of the patients with bladder tumours were analysed by Western blotting following electrophoresis on 8% sodium dodecyl sulphate (SDS) polyaryclamide gels under reducing conditions, as previously described (Banks et al, 1995).

**Table 1** Details of the sample groups

| | Healthy controls | Benign group | Cancer group | Total number |
|---|---|---|---|---|
| Total number | 21 | 14 | 34 | 69 |
| Male | 10 | 9 | 21 | 40 |
| Female | 11 | 5 | 13 | 29 |
| Age range (years) | 30–82 | 16–85 | 44–89 | 16–89 |
| Mean age (years) | 56.1 | 65.9 | 70.8 | |

**Table 2** Stage/grade of bladder tumours

| | Number of patients |
|---|---|
| Tumour grade | |
| G1 | 4 |
| G2 | 17 |
| G3 | 13 |
| Tumour stage | |
| pTa | 23 |
| pT1 | 3 |
| pT2 | 0 |
| pT3 | 7 |
| pT4 | 1 |

### Statistical analysis

Statistical analysis was carried out using SPSS-PC (SPSS Inc, Illinois, USA). As the data were found to be non-normally distributed using the Shapiro–Wilks and K–S (Lilliefors) tests, the non-parametric Mann–Whitney test and Spearman's correlation were used. Differences were judged to be statistically significant if $P < 0.05$.

### RESULTS

A total of 69 samples were assayed. As the ages between the normal (healthy and benign controls) and malignant groups differed significantly ($P < 0.05$), the relationship of the assayed parameters with age was examined but no correlations were found.

Considerable overlap was seen between the groups regarding urinary sE-cadherin concentrations (Figure 1A) with the only statistically significant differences being seen between the control (range 24.9–1437, median 582) and neoplastic group, whether the latter was examined as a whole (range 12–3813, median 1272.5) or subdivided into the non-invasive (pTa; range 12–3812, median 1284) and invasive (pT1–4; range 97.7–2327.2, median 1551) cancers ($P < 0.001$, $P < 0.01$, $P < 0.01$ respectively). When adjusted for creatinine concentration (Figure 1B), the difference between the control (range 0.058–1.481, median 0.516) and neoplastic groups (whole: range 0.043–6.0, median 1.536; pTa: range 0.043–5.271, median 1.477; pT1–4: range 0.026–6.0,

© Cancer Research Campaign 1999

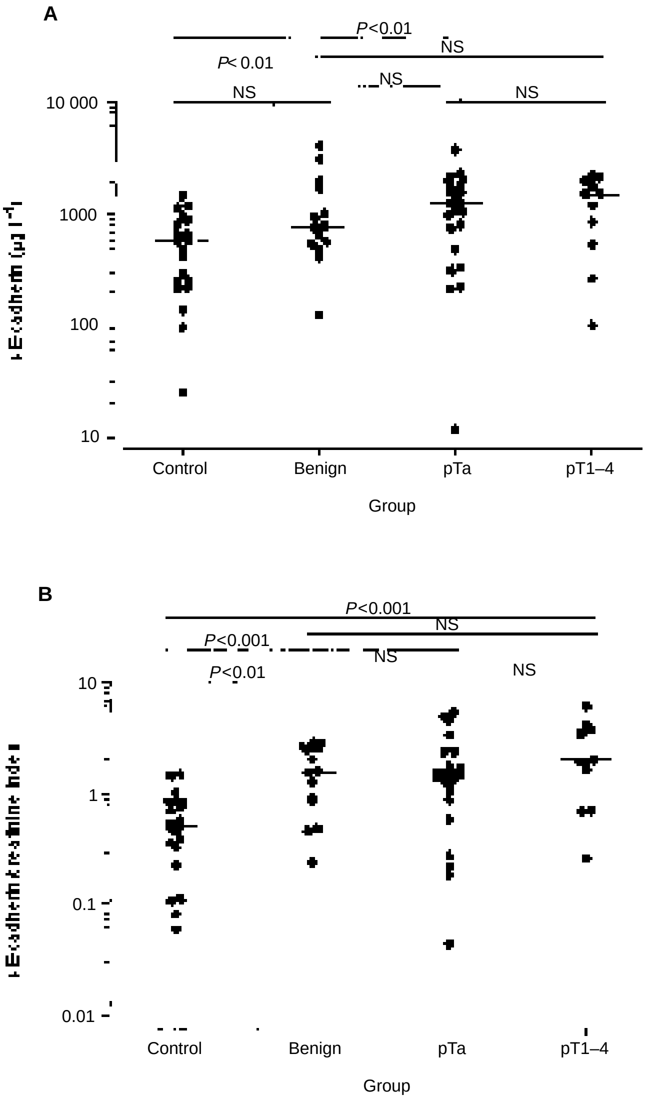

**Figure 1** (**A**) Urinary sE-cadherin levels (µg l⁻¹) and (**B**) urinary sE-cadherin/creatinine index versus patient group; — represents the median value

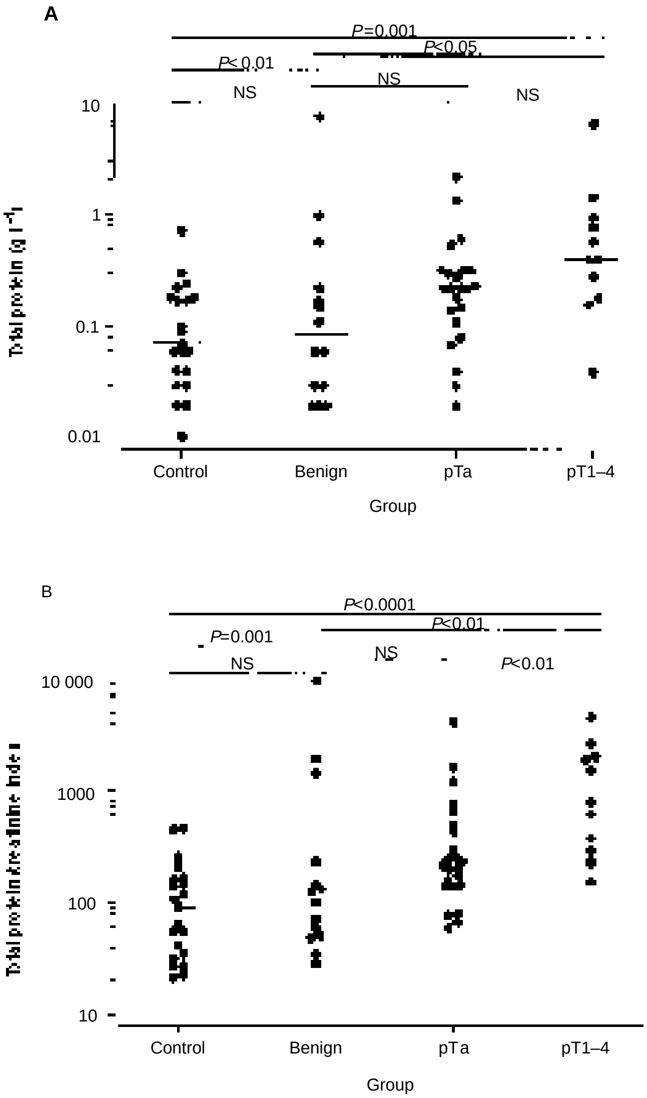

**Figure 2** (**A**) Urinary total protein (g l⁻¹) and (**B**) protein/creatinine index versus patient group;— represents the median value median 1.939) became more significant ($P$ = 0.0001, $P$ < 0.001 and $P$ < 0.001 respectively) and additionally a significant difference ($P$ < 0.01) was seen between the control (range 5.8–148.1, median 51.6) and benign group (range 24.1–282.6, median 157.3). However, there was no apparent difference between the benign group and patients with cancer, or between patients with non-invasive (pTa) or invasive disease (pT1–4).

When the urinary total protein concentrations were analysed (Figure 2A), considerable overlap between groups was again seen, but patients with tumours (range 0.03–6.38, median 0.255) had significantly higher concentrations than those of the control group (range 0.01–0.72, median 0.07) ($P$ < 0.001) or the benign group (range 0.02–7.75, median 0.085) ($P$ < 0.05). However, when the neoplastic group was subdivided into invasive (pT1–4) (range 0.04–6.38, median 0.4) and non-invasive (pTa) cases (range 0.02–2.19, median 0.22), only the protein concentrations of the invasive sub-group were significantly higher than those of the benign group ($P$ < 0.05). No significant difference was seen between the invasive and non-invasive groups or between the benign and control groups. When the protein/creatinine index was used (Figure 2B), similar but more significant differences were seen between the groups (healthy controls: range 20.8–454.6, median 90.9; benign controls: range 27.8–10 616.4, median 113.2; neoplastic group: range 59.7–4524.8, median 263.6) and, in addition, a significant difference ($P$ < 0.01) was seen between

**Table 3** Numbers of urine samples with each form of sE-cadherin as detected by Western blotting

| sE-cadherin | Normal controls ($n$ = 12) | Cancer group ($n$ = 21) | | | | | | | |
|---|---|---|---|---|---|---|---|---|---|
| | | Grade | | | Stage | | | | |
| | | G1 | G2 | G3 | pTa | pT1 | pT2 | pT3 | pT4 |
| No band | 1 | 0 | 1 | 1 | 1 | 0 | 0 | 1 | 0 |
| 80 kDa only | 8 | 1 | 9 | 5 | 11 | 1 | 0 | 2 | 1 |
| 80 and 65 kDa | 3 | 0 | 1 | 3 | 2 | 1 | 0 | 1 | 0 |

*© Cancer Research Campaign 1999*

non-invasive (range 59.7–4206.9, median 214.3) and invasive cancers (range 153.9–4524.8, median 776).

No significant correlation was found between sE-cadherin concentrations (absolute or following adjustment for creatinine) or total protein and tumour stage or grade. However, the protein/creatinine index correlated significantly with tumour stage ($P < 0.01$) and just failed to reach a significant correlation with tumour grade ($P < 0.06$).

A main sE-cadherin band of 80–85 kDa was detected by Western blotting (as shown previously; Banks et al, 1995) in 30 out of 33 samples (Table 3) with a further additional band of 65–70 kDa apparent in 20–25% of both the control and patient samples. No bands were detected in the blots incubated with an irrelevant primary antibody as a non-specific binding control.

### DISCUSSION

Loss or decreased expression of the adhesion molecule E-cadherin correlating with tumour progression has now been shown immunohistochemically in many tumour types (reviewed by Takeichi, 1993) illustrating the important role of this molecule in maintaining normal epithelial cohesion. Earlier studies showing the occurrence of a soluble form of this molecule in cell culture systems in vitro (Damsky et al, 1983) have led to the search for and subsequent finding of soluble forms in biological fluids in vivo with the hope that such a molecule may in some way reflect tumour progression and hence lend itself to being a tumour marker. Following our previous observations of the presence of sE-cadherin in urine (Banks et al, 1995), we have now demonstrated that urinary sE-cadherin concentrations are significantly increased in bladder cancer patients compared to normal controls, whether expressed as an absolute concentration or following adjustment for creatinine concentration.

The exact mechanism of production of the soluble form of E-cadherin is not known. Alternatively spliced forms of some adhesion molecules such as P-selectin have been described which lack the transmembrane region (Gearing and Newman, 1993) but similarly generated transcripts have not been described as yet for soluble E-cadherin. Proteolytic cleavage is also known to be important in the generation of many soluble molecules and studies have shown such a mechanism can occur for soluble E-cadherin (Damsky et al, 1983; Katayama et al, 1994*a*) with the intact molecule of 110–120 kDa being proteolytically digested to yield an 80 kDa molecule as found in this study. This may occur to a limited extent during normal cellular turnover but would be expected to be increased in conditions where protease release is enhanced. As such, significant differences in sE-cadherin concentrations would be expected between the non-invasive and invasive subgroups. The apparent absence of any such difference may be due to the relatively small number of patients in this study, although considerable overlap is seen between the groups. Given the overlap between groups, and the lack of correlation with stage or grade, the clinical use is likely to be limited.

The existence of two soluble forms of E-cadherin in urine (65 kDa and 80 kDa) as detected by Western blotting was described in our original study (Banks et al, 1995) and confirmed in this study. The significance of the two forms is unclear with the simultaneous presence of both forms being seen in 20–25% of both controls and patients with bladder cancer. It is likely that proteolysis accounts for the two fragments of the protein but this needs to be confirmed. The proteolysis is unlikely to have occurred in vitro as samples were collected containing protease inhibitors. It is also not known whether both these fragments are detected by the immunoassay used in this study which may result in underestimation of the sE-cadherin concentration in some samples. Soluble E-cadherin molecules with electrophoretic mobilities ranging from 75 to 85 kDa have been reported before in urine samples from patients with benign and malignant conditions (Katayama et al, 1994*b*).

The only other study which has examined sE-cadherin concentrations in patients with bladder cancer analysed serum samples from patients at presentation (Griffiths et al, 1996) and found significantly higher concentrations than controls in patients with high-grade tumours or muscle-invasive disease. These are similar to our findings using urine samples, although we detected a significant difference between the control and non-invasive groups. No apparent difference was seen between the non-invasive and invasive groups in either study and neither did serum sE-cadherin concentration correlate with abnormal E-cadherin expression on the surface of bladder tumours (Griffiths et al, 1996). This does not support an increased rate of shedding as being the sole mechanism for the decreased cellular expression in cancer.

Increased urinary protein excretion is a relatively common finding in patients with bladder cancer, with concentration being related to tumour size but not grade and a significant reduction being seen following successful treatment (Johansson, 1975; Johansson and Kistner, 1975; Hemmingsen et al, 1981). Qualitative studies have shown the increased concentration of a number of proteins including IgG, IgA, transferrin, $\alpha_1$-acid glycoprotein, haptoglobin, fibrinogen, $\alpha_1$-antitrypsin and $\alpha_2$-macroglobulin, a similar profile also being seen in infection (Johansson, 1975; Johansson and Kistner, 1975; O'Brien et al, 1980, 1981). The finding of a predominantly glomerular proteinuria decreasing, but not returning to normal, after successful treatment or urinary diversion, particularly in higher grade tumours where it may be of predictive value (Hemmingsen et al, 1981), has been proposed to be associated with the finding of glomerular deposition of antigen–antibody complexes in urothelial malignancies (Jones et al, 1975). Additionally, some proteins may be present due to leakage from the plasma, possibly caused by tumour neovascularization, invasion or an inflammatory response, or alternatively may be derived from the tumour itself as a result of increased cellular turnover. Examining total protein concentration provides limited information due to its lack of tumour-specificity, being elevated, for example, by infection. However, in this study at least, total protein appears to be superior to sE-cadherin as a 'marker' in bladder cancer and is considerably cheaper to assay. Although the protein/creatinine ratio is significantly different between groups, the considerable overlap prohibits its use diagnostically. It is possible that longitudinal measurements may be of use on an individual patient basis as an initial follow-up screen but additional factors producing an increase in protein concentrations such as infection would need to be excluded.

Expressing the urinary results following adjustment for creatinine concentration is probably the more valid reflection of overall sE-cadherin or total protein release as it takes into account the variations in the renal function and hydration state of patients and when evaluated for total protein measurements has been shown to allow the use of single sample analysis as an adequate substitute

© Cancer Research Campaign 1999

for 24-h collection and measurement (Dyson et al, 1992; Mitchell et al, 1993). Whether there is a diurnal variation on sE-cadherin concentration, and thus an optimal time for sample collection other than the mid-morning samples used in this study, was not established. The presence of intercurrent renal disease should also be taken into account due to its effects on the creatinine and hence the creatinine adjusted parameters. In this study, none of our patients were being treated for renal disease although six patients had mildly elevated serum creatinine concentrations.

Although significant differences were found between normal control and malignant groups, the overlap in the values between these groups and also those of the benign group indicate that measurement of urinary sE-cadherin concentrations alone could not be used as the basis for screening for malignant disease. However, in the light of the earlier results showing patients at risk of early relapse had significantly elevated levels of serum sE-cadherin at presentation (Griffiths et al, 1996), this should be explored further in a longitudinal study using both serum and urine samples. In addition, the use of urinary total protein should be explored further as it appears to be superior to sE-cadherin in its discriminatory capacity and would be more cost effective.

### REFERENCES

Banks RE, Porter WH, Whelan P, Smith PH and Selby PJ (1995) Soluble forms of the adhesion molecule E-cadherin in urine. *J Clin Path* **48**: 179–180

Becker KF and Hofler H (1995) Frequent somatic allelic inactivation of the E-cadherin gene in gastric carcinomas. *J Natl Cancer Inst* **87**: 1082–1084

Bringuier PP, Umbas R, Schaafsma HE, Karthaus HFM, Debrugne FMJ and Schalken JA (1993) Decreased E-cadherin immunoreactivity correlates with poor survival in patients with bladder tumours. *Cancer Res* **53**: 3241–3245

Caliskan M, Turkeri LN, Mansuroglu B, Toktas G, Aksoy B, Uncluer E and Akdas A (1997) Nuclear accumulation of mutant p53 protein: a possible predictor of failure of intravesical therapy in bladder cancer. *Br J Urol* **79**: 373–377

*Cancer Research Campaign Factsheet 9.1* (1997) Cancer Research Campaign: London

Damsky CH, Richa J, Solter D, Knudsen K and Buck CA (1983) Identification and purification of a cell surface glycoprotein mediating intercellular adhesion in embryonic and adult tissue. *Cell* **34**: 455–466

D'Hallewin MA and Baert L (1996) Initial evaluation of the bladder tumour antigen test in superficial bladder cancer. *J Urol* **155**: 475–476

Dyson EH, Will EJ, Davidson AM, O'Malley AH, Shepard HT and Jones RG (1992) Use of the urinary protein creatinine index to assess proteinuria in renal transplant patients. *Nephrol Dial Transplant* **7**: 450–452

Esrig D, Elmajian D, Groshen S, Freeman J, Stein J, Chen SC, Nichols P, Skinner D, Jones P and Cote R (1994) Accumulation of nuclear p53 and tumour progression in bladder cancer. *N Engl J Med* **331**: 1259–1264

Gearing AJH and Newman W (1993) Circulating adhesion molecules in disease. *Immunol Today* **14**: 506–512

Gohji K, Fujimoto N, Ohkawa J, Fujii A and Nakajima M (1998) Imbalance between serum matrix metalloproteinase-2 and its inhibitor as a predictor of recurrence of urothelial cancer. *Br J Cancer* **77**: 650–655

Griffiths TRL, Brotherick I, Bishop RI, White MD, McKenna DM, Horne CHW, Shenton BK, Neal DE and Mellon JK (1996) Cell adhesion molecules in bladder cancer: soluble serum E-cadherin correlates with predictors of recurrence. *Br J Cancer* **74**: 579–584

Hemmingsen L, Rasmussen F, Skaarup P and Wolf H (1981) Urinary protein profiles in patients with urothelial bladder tumours. *Br J Urol* **53**: 324–329

Hermanek P and Sobin LH (eds) (1987) UICC *TNM Classification of Malignant Tumours*, 4th edn, pp. 65–67. Springer Verlag: Berlin

Hiraguri S, Godfrey T, Nakamura H, Graff J, Collins C, Shayesteh L, Doggett N, Johnson K, Wheelock M, Herman J, Baylin S, Pinkel D and Gray J (1988) Mechanisms of inactivation of E-Cadherin in breast cancer cell lines. *Cancer Res* **58**: 1972–1977

Hudson MA and Catalona WJ (1996) Urothelial tumours of the bladder, upper tracts and prostate. In *Adult and Paediatric Urology*, Gillenwater JY, Grayhack JT, Howards SS and Duckett JW (eds), pp. 643–694. Mosby: St Louis, MO

Johansson B (1975) Urinary protein patterns in patients with uroepithelial tumours. *Scand J Urol Nephrol* **9**: 221–225

Johansson B and Kistner S (1975) Proteinuria in patients with uroepithelial tumours with special regard to tumour size, clinical staging and grade of malignancy. *Scand J Urol Nephrol* **9**: 45–49

Jones LW, Levin A and Fudenberg HH (1975) Glomerular antigen complexes associated with transitional cell carcinoma. *Surg Gynecol Obstet* **140**: 896–898

Kanai Y, Oda T, Tsuda H, Ochiai A and Hirohashi S (1994) Point mutation of the E-cadherin gene in invasive lobular carcinoma of the breast. *Jpn J Cancer Res* **85**: 1035–1039

Kanayama H, Yokota K, Kurokawa Y, Murakami Y, Nishitani M and Kagawa S (1998) Prognostic values of matrix metalloproteinase-2 and tissue inhibitor of metalloproteinase-2 expression in bladder cancer. *Cancer* **82**: 1359–1366

Katayama M, Hirai S, Kamihagi K, Nakagawa K, Yasumoto M and Kato I (1994*a*) Soluble E-cadherin fragments increased in circulation of cancer patients. *Br J Cancer* **69**: 580–585

Katayama M, Hirai S, Yasumoto M, Nishikawa K, Nagata S, Otsuka M, Kamihagi K and Kato I (1994*b*) Soluble fragments of E-cadherin cell adhesion molecule increase in urinary excretion of cancer patients, potentially indicating its shedding from epithelial tumour cells. *Int J Oncol* **5**: 1049–1057

Kinoshita H, Ogawa O, Kakehi Y, Mishina M, Mitsumori K, Itoh N, Yamada H, Terachi T and Yoshida O (1997) Detection of telomerase activity in exfoliated cells in urine from patients with bladder cancer. *J Natl Cancer Inst* **89**: 724–730

Leyh H, Hall R, Mazeman E and Blumenstein B (1997) Comparison of the BARD BTA test with voided urine and bladder wash cytology in the diagnosis and management of cancer of the bladder. *Urology* **50**: 49–53

Lipponen PK and Eskelinen MJ (1995). Reduced expression of E-cadherin is related to invasive disease and frequent recurrence in bladder cancer. *J Cancer Res Clin Oncol* **121**: 303–308

Mao L, Schoenberg M, Scicchitano M, Erozan Y, Merlo A, Schwab D and Sidransky D (1996) Molecular detection of primary bladder cancer by microsatellite analysis. *Science* **271**: 659–662

Matsuura K, Kawanishi J, Fujii S, Imamura M, Hiranos S, Takeichi M and Niitsu Y (1992) Altered expression of E-cadherin in gastric cancer tissue and carcinomatous fluid. *Br J Cancer* **66**: 1122–1130

Mitchell S, Sheldon T and Shaw A (1993) Quantification of proteinuria: a re-evaluation of the protein/creatinine ratio for elderly subjects. *Age Ageing* **22**: 443–449

Mostofi FK, Sobin LH and Torloni H (1973) Histological typing of urinary bladder tumours. In *International Histological Classification of Tumours, 10*: World Health Organization: Geneva

O'Brien P, Gozzo J, Cronin W and Monaco A (1980) Qualitative analysis of proteinuria associated with bladder cancer. *Invest Urol* **17**: 28–32

O'Brien P, Gozzo JJ and Monaco AP (1981) Urinary proteins as biological markers: bladder cancer diagnosis versus urinary tract infection. *J Urol* **124**: 802–803

Otto T, Birchmeier W, Schmidt U, Hinke A, Schipper J, Rubben H and Raz A (1994) Inverse relation of E-cadherin and autocrine motility factor receptor expression as a prognostic factor in patients with bladder carcinomas. *Cancer Res* **54**: 3120–3123

Ozawa M, Engel J and Kemler R (1990) Single amino acid substitutions in one $Ca^{2+}$ binding site of uvomorulin abolish adhesive function. *Cell* **63**: 1033–1038

Parmar MKB, Freedman LS, Hargreave TB and Tolley DA (1989) Prognostic factors for recurrence and follow-up policies in the treatment of superficial bladder cancer: report from the British Medical Research Council Subgroup on Superficial Bladder Cancer (Urological Cancer Working Party). *J Urol* **142**: 284–288

Reading J, Hall RR and Parmar MKB (1995) The application of a prognostic factor analysis for Ta.T1 bladder cancer in routine urological practice. *Br J Urol* **75**: 604–607

Rock RC, Walker WG and Jennings CD (1986) Nitrogen metabolites and renal function. In *Textbook of Clinical Chemistry*, Tietz NW (ed), pp. 1254–1316. WB Saunders: Philadelphia

Ross JS, Del Rosario AD, Figge HL, Sheehan C, Fisher HAG and Bui HX (1995) E-cadherin expression in papillary transitional cell carcinoma of the urinary bladder. *Hum Pathol* **26**: 940–944

Sarosdy M, deVERE White R, Soloway M, Sheinfeld J, Hudson M, Schellhammer P, Jarowenko M, Adams G and Blumenstein B (1995) Results of a multicentre trial using the BTA test to monitor for and diagnose recurrent bladder cancer. *J Urol* **154**: 379–384

Shimoyama Y, Nagafuchi A, Fujita S, Gotoh M, Takeichi M, Tsukita S and Hirohashi S (1992) Cadherin dysfunction in a human cancer cell line: possible involvement of loss of α-catenin expression in reduced cell–cell adhesiveness. *Cancer Res* **52**: 5770–5774

Soloway M, Briggman J, Carpinito G, Chodak G, Church P, Lamm D, Lange P, Messing E, Pasciak R, Reservitz G, Rukstalis D, Sarosdy M, Stadler W, Thiel R and Hayden C (1996) Use of a new tumour marker, urinary NMP22, in the

© Cancer Research Campaign 1999

detection of occult or rapidly recurring transitional cell carcinoma of the urinary tract following surgical treatment. *J Urol* **156**: 363–367
Steiner G, Schoenberg MP, Linn JF, Mao L and Sidransky D (1997) Detection of bladder cancer recurrence by microsatellite analysis of urine. *Nature Med* **3**: 621–624
Syrigos K, Krausz T, Waxman J, Pandha H, Rowlinson-Busza G, Verne J, Epenetos A and Pignatelli M (1995). E-cadherin expression in bladder cancer using formalin-fixed, paraffin-embedded tissues: correlation with histopathological grade, tumour stage and survival. *Int J Cancer* **64**: 367–370
Takeichi M (1990) Cadherins: a molecular family important in selective cell–cell adhesion. *Ann Rev Biochem* **59**: 237–252
Takeichi M (1993) Cadherins in cancer implications for invasion and metastasis. *Curr Opin Cell Biol* **5**: 806–811
Tsuji M, Kojima Y, Murakami Y, Kanayama H and Kagawa S (1997) Prognostic value of Ki-67 antigen and p53 protein in urinary bladder cancer: immunohistochemical analysis of radical cystectomy specimens. *Br J Urol* **79**: 367–372
Yoshida K, Sugino T, Tahara H, Woodman A, Bolodeoku J, Nargund V, Fellows G, Goodison S, Tahara E and Tarin D (1997) Telomerase activity in bladder carcinoma and its implication for noninvasive diagnosis by detection of exfoliated cancer cells in urine. *Cancer* **79**: 362–369
Yoshiura K, Kanai Y, Ochiai A, Shimoyama Y, Sugimura T and Hirohashi S (1995) Silencing of the E-cadherin invasion-suppressor gene by CpG methylation in human carcinomas. *Proc Natl Acad Sci USA* **92**: 7416–7419

*© Cancer Research Campaign 1999*
